# Enhancing the Compatibility, Hydrophilicity and Mechanical Properties of Polysulfone Ultrafiltration Membranes with Lignocellulose Nanofibrils

**DOI:** 10.3390/polym8100349

**Published:** 2016-10-14

**Authors:** Zhaodong Ding, Xuejiao Liu, Yang Liu, Liping Zhang

**Affiliations:** Ministry of Education Engineering Research Center of Forestry Biomass Materials and Bioenergy, Department of Material Science and Technology, Beijing Forestry University, Beijing 100083, China; dzdhero@163.com (Z.D.); liuxuejiao@bjfu.edu.cn (X.L.); lyceum525@163.com (Y.L.)

**Keywords:** polysulfone, lignocellulose nanofibrils, ultrafiltration membranes

## Abstract

Lignocellulose nanofibrils (LCN) and cellulose nanofibrils (CNF) are popular nanometer additives to improve mechanical properties and hydrophilic abilities; moreover, lignocellulose has potential as a natural adhesion promoter in fiber-reinforced composites. LCN and CNF were blended into polysulfone (PSF) to prepare ultrafiltration membranes via the phase inversion method. These additives were characterized by Fourier transform infrared spectroscopy and transmission electron microscopy, and the rheological properties such as shear viscosity and non-Newtonian fluid index of the casting solutions were analyzed using a rotational rheometer. The performance of ultrafiltration membranes was characterized using Fourier transform infrared spectroscopy, thermogravimetric analysis and scanning electron microscopy. The pure water flux, bovine serum albumin retention ratio, water contact angle, surface energy, molecular weight cut-off, pore size and mechanical properties were measured. The equilibrium contact angle of water decreased from 63.5° on the PSF membrane to 42.1° on the CNF/PSF membrane and then decreased to 33.9° on the LCN/PSF membrane when the nanometer additives content was 0.8 wt %. The results reveal that LCN and CNF were successfully combined with PSF. Moreover, the combination of LCN/PSF ultrafiltration membranes was more promising than that of CNF/PSF ultrafiltration membranes.

## 1. Introduction

Ultrafiltration membranes have an important status in industry, and are used in a wide range of applications [1]. Polysulfone (PSF) is an extensively used ultrafiltration membrane material that has been widely applied in water treatment, biomedical applications and other industrial fields [2,3,4,5,6,7,8,9]. PSF contains repeated ether and sulfone linkages alternating between aromatic rings, and it is reliably available with qualities such as high chemical and hydrolytic stability as well as film-forming properties [10,11,12]. However, fouling of polymeric membranes is a problem in membrane separation processes [13,14,15].

Different strategies have been carried out to prevent membrane biofouling. Increasing the hydrophilicity can be done by increasing the amount of hydrophilic functional groups on the membrane surface [16,17]. Physical blending is an effective method to reduce membrane biofouling due to the introduction of anti-fouling materials to the membrane matrix [18,19,20]. The advantages of physical blending are that it is less complicated than developing new polymerizations, carried out under mild conditions and inexpensive [21,22]. Using unbleached wood pulp and bleached wood pulp to produce Lignocellulose nanofibrils (LCN) and cellulose nanofibrils (CNF) that have nanosized fiber diameters show better hydrophilic performance and mechanical properties than a majority of widely used reinforcement materials. LCN and CNF are hydrophilic and reinforced materials, and they are able to improve the hydrophilicity and mechanical properties of PSF ultrafiltration membranes by blending in the casing solution. LCN as a chain extender is able to promote compatibility between cellulose and the polymer matrix [23]. Many researchers have studied the effects of additives with hydrophilic properties on the membrane morphology and performance. However, applying LCN as membrane additives is a new addition to the array of available strategies for increasing the hydrophilicity of membranes.

LCN consists of lignin as a natural adhesion promoter and cellulose as hydrophilic chain segment. A mass of lignin–carbohydrate complexes exist between lignin and cellulose, which ensures the stability of LCN. Lignin, a three-dimensional amorphous polymer consisting of methylated phenylpropane structures, is interesting because it is a waste product from the paper industry [24]. Only a small part of this amorphous poly macromolecule is used in material application [25,26]. Previous work has shown that lignin can be used as an additive in blended fabrications [27]. Lignin was applied as a natural adhesion promoter in biodegradable and thermoplastic cotton fiber-reinforced composites by Grainer et al. [28]. Lignin was adopted by Beget et al. [29] to reinforce polypropylene composites. LCN was used as a reinforced material to blend with PLA by Wang et al. [30]. Lignin as a hydrophobic chain segment may provide an adhesion modification effect, because it can anchor to the membrane matrix through supramolecular non-covalent interactions. Therefore, lignin as a natural adhesion promoter may improve LCN adhesion in the membrane matrix and enhance the hydrophilic and mechanical abilities of PSF/LCN blend ultrafiltration membranes. Cellulose is an attractive renewable and biodegradable resource composed of poly-(1,4)-d-glucose residues, and its hydrophilic nature allows water adsorption [31,32]. Cellulose is a preferred additive for blending because of its good mechanical strength, flexibility and low cost [33,34,35]. The specific control of interactions between cellulose and polymer matrices is relevant to designing hydrophilic membranes [36,37].

**Figure 1:**
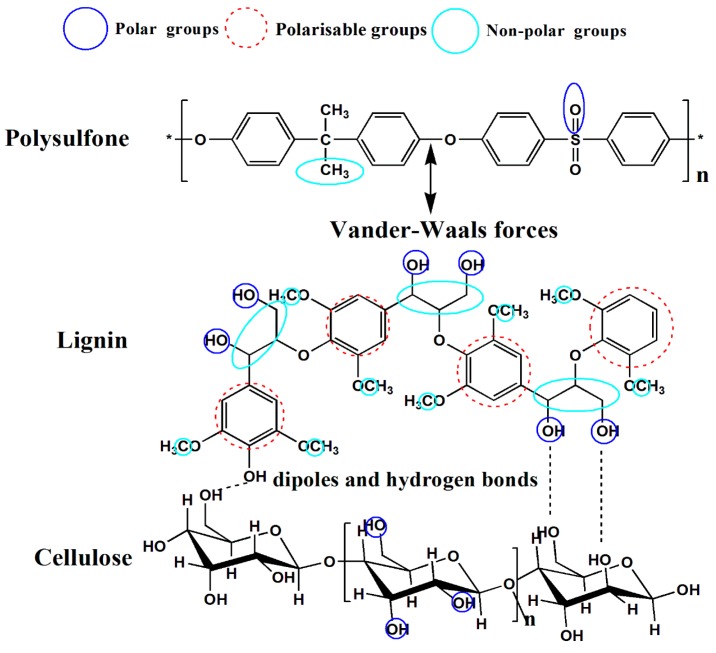
The absorption promoter mechanism of lignin on cellulose adhesion to the membrane matrix.

This study investigated how lignin—used as a natural adhesion promoter in cellulose fiber-reinforced composites—influence the ultrafiltration membranes properties. LCN as a hydrophilic additive and mechanical reinforcement material was selected to blend with PSF in a casting solution. Because of the absorption promoter effect of lignin on cellulose adhesion to the membrane matrix, more hydrogen bonds can form between the –OH groups of cellulose and the sulfone groups of PSF, and LCN can firmly assemble within the membrane matrix. In the phase inversion process, the hydrophilic layer is built [38]. The effects of LCN concentration on morphology and membrane performance are investigated. It is the first time to study the absorption promoter mechanism of lignin on cellulose adhesion to the PSF membrane matrix, which is shown in Figure 1, compared to our previous work [38]. Moreover, we still need more work to investigate three-dimensional structure of lignocellulose and compatibility mechanism of lignin on cellulose adhesion to the polymer matrix.

## 2. Experimental

### 2.1. Materials

PSF and polyethylene glycol (PEG) were purchased from Dongguan City Special Exhibition of Plastic Raw Material Co., Ltd. (Dongguan, China). Polyvinylpyrrolidone (PVP) as pore-foaming agent and bovine serum albumin (BSA) were purchased from Shantou Exiling Chemical Plant (Shantou, China) and Beijing Aoboxing Biological Technology Co., Ltd. (Beijing, China), respectively. Unbleached wood pulp and bleached wood pulp were provided by Shandong Huatai Paper (Dongying, China). H_2_SO_4_ (95%–98%) and *N*,*N*-dimethylacetamide were purchased from Beijing Chemical Plant (Beijing, China).

### 2.2. Preparation of CNF and LCN

Bleached wood pulp and unbleached wood pulp (lignin content was 9.3%) was immersed into H_2_SO_4_ (20 wt %) solution (solid–liquid rate was 1:20) and reacted at 85 °C by mixing sufficiently with an electric blender (6 h). At the end of the reaction, the pH value of the solution was increased and washed using deionized water and centrifugal machine, until it was neutral. After offcentering and sieving, the solids were submerged into *N*,*N*-dimethylacetamide and homogenized with a high-pressure homogenizer (GEA, Parma, Italy) at 1000 bar. A colloidal suspension of CNF or LCN was obtained, and the lignin content of LCN was 13.8%. CNF and LCN were diluted with *N*,*N*-dimethylacetamide to varying weight percentages, 0, 0.2, 0.4, 0.6, 0.8, 1 and 1.2 wt %, to study the effects of the CNF or LCN concentration on the PSF ultrafiltration membrane performance.

### 2.3. Preparation of Casting Solution

A quantity of PSF and additives were dissolved in a LCN or CNF colloidal suspension at different concentrations (Table 1). The solution was obtained by shaking (60 r/min) at 50 °C (Constant-temperature table concentrator, SHK-99-II, Beijing North TZ-Biotech Develop Co., Beijing, China) for 24 h. After vacuum degassing, the solution was obtained.

### 2.4. Preparation of the Blend Membranes

**Figure 2:**
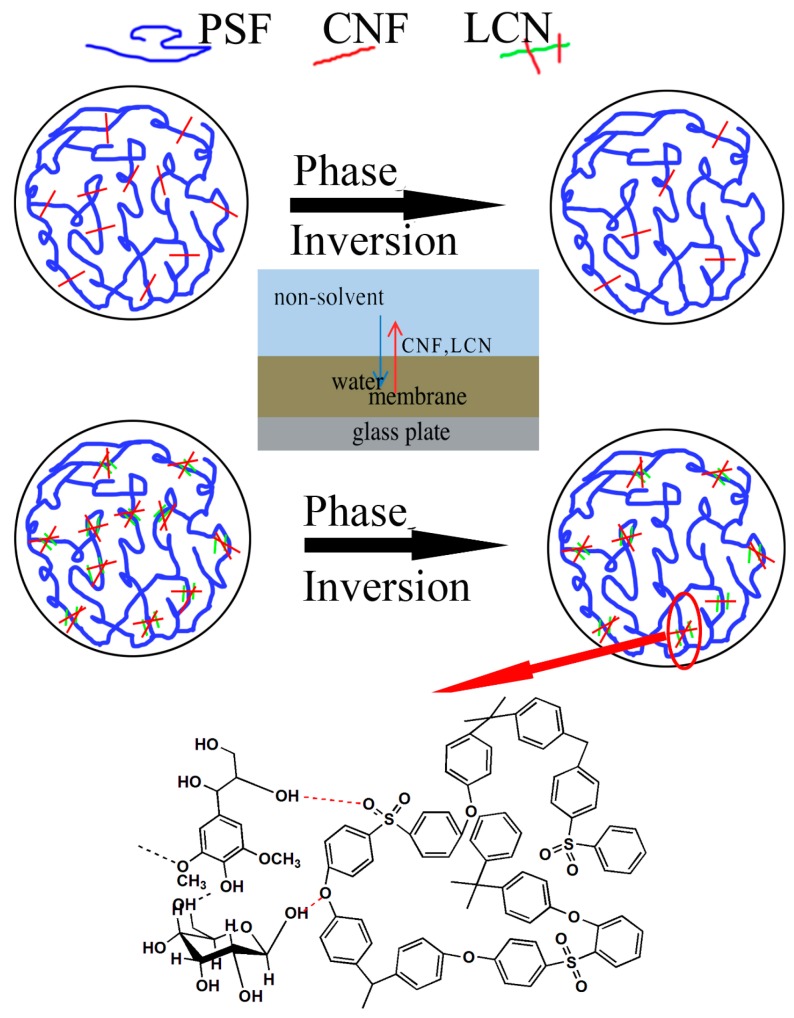
The possible membrane formation mechanism affected by the cellulose nanofibrils (CNF) and Lignocellulose nanofibrils (LCN).

The possible membrane formation mechanism affected by the CNF and LCN is presented in Figure 2. Membranes were prepared via the phase inversion method. A quantity of PSF (18 wt %) and polyvinylpyrrolidone (0.3 wt %) were dissolved in the prepared CNF or LCN colloidal suspension (in different concentrations). The casting solution was obtained after shaking at 60 °C for 24 h using a table concentrator (SHK-99-II, Beijing North TZ-Biotech Develop Co., Beijing, China). After vacuum degassing, an appropriate amount of the solution was cast on a glass plate at ambient conditions. After exposure in air for 10 s, the glass plate was immersed into a water bath at room temperature. After coagulation was complete, the membranes were washed in water and used for characterization.

### 2.5. Ultrafiltration Experimental Setup

The ultrafiltration experiments were carried out in a batch-type dead end cell (UF cell, Model 8400, Amicon, THERMO NALGENE, New York, NY, USA) with an internal diameter of 76 mm and fitted with a Teflon-coated magnetic paddle. The effective membrane area available for ultrafiltration was 38.5 cm^2^.

### 2.6. Characterization

#### 2.6.1. Morphology Observations of CNF, LCN and Membranes

CNF or LCN was examined with a Hitachi H-600 transmission electron microscope (TEM, HITACHI, Tokyo, Japan) at an acceleration voltage of 80 kV. To examine the CNF or LCN, one droplet of 0.01% diluted suspension was put on a Cu grid. To enhance contrast in TEM, the CNF or LCN was negatively stained with a 2 wt % aqueous solution of phosphotungstic acid for 1 min.

The cross-sectional morphologies of the membranes were investigated by scanning electron microscopy (SEM; LEO 1550, with a 5-kV Schottky field emission gun and a Robinson backscatter detector, HITACHI, Tokyo, Japan). All specimens received 45 s of gold coating. The cross-sectional view of the sample was obtained by fracturing the water-wetted membrane in liquid nitrogen.

#### 2.6.2. Structure Characteristic of the CNF, LCN and the Membranes

FTIR of the CNF, LCN and the membranes were recorded using an ATR-FTIR spectrometer (UATR, Perkin Elmer, Waltham, MA, USA) in the range of 500–4000 cm^−1^.

#### 2.6.3. Rheological Performance of Membrane Casting Solution

The shear viscosity and non-Newtonian fluid index were studied with a Rotational Rheometer (CVO-100-901, Malvern, Melvin, UK) equipped with 25-mm diameter stainless steel cylinder and parallel discs.

#### 2.6.4. Shear Viscosity

The shear viscosity of all the casting solution was measured at 25 °C. The steady-state test mode was at a shear rate 10 s^−1^.

#### 2.6.5. Non-Newtonian Fluid Index

The shear viscosity of all the casting solution was measured at 25 °C. The shear rate ranged from 0.5 to 1000 s^−1^. T shear stress-shear rate was obtained. The non-Newtonian fluid index was calculated using the following equation:

(1) $$\sigma =K\cdot {\dot{\gamma }}^{n}$$

where σ is the shear stress (Pa), $\dot{\gamma }$ is the shear rate (s^−1^) and *n* is the non-Newtonian fluid index.

#### 2.6.6. Thermal Ability of CNF, LCN and Membranes

The thermogravimetric analysis (TGA) and differential scanning calorimetry (DSC) of CNF, LCN and the membranes was carried out in a Universal V4.5A TA DTG analyzer (HITACHI, Tokyo, Japan) in a nitrogen atmosphere. The samples were heated from room temperature to 600 °C at a heating rate of 20 °C/min. From the TGA, the thermal degradation characteristics such as onset of degradation (*T*_on_), temperature of maximum rate of degradation (*T*_max_), glass-transition temperature (*T*_g_) and percentage weight losses at different temperatures were calculated.

#### 2.6.7. Mechanical Properties of Membranes

To evaluate the mechanical properties of membranes, a tensile testing machine (DCP-KZ300, Dong Ao, Chengdu, China) was used to test the tensile strength, breaking elongation and Young’s modulus of membranes. The speed of cross head was 20 mm/min. The dried membranes were snipped into a rectangle with a width of 15 mm, a length of 100 mm and a thick of 0.2 mm. All membranes were tested in ambient conditions.

#### 2.6.8. Pure Water Flux (PWF) and Bovine Serum Albumin (BSA) Rejection Ratio of Membranes

The flux was measured using the method outlined in previous works [39]. The pure water flux (PWF) was measured by an ultrafiltration experimental setup. The initial water flux was taken about 30 min after pressurization in the ultrafiltration apparatus and working at 0.1 MPa during the test. PWF (*J*_w_) was calculated at time intervals using the following equation:

*J*_w_ = *Q*/(*A* × Δ*T*) (2)

where *Q* is permeating of pure water (L), *A* is the effective membrane area (m^2^) and Δ*T* (h) is the sampling time.

The retention coefficient (*R*) of membranes was measured by calculating the fluid retention capacity of BSA (5.0 × 10^4^ Da) through the membrane. A UV spectrophotometer (UV-1801, BFRL, Beijing, China) was used to measure the absorbance of the BSA solution (1 g/L) and the permeate solution at 280 nm. All tests were conducted at a working pressure of 0.1 MPa and room temperature. The retention coefficient (*R*) was calculated as follows:

(3) $$R(\%)=(1-\frac{A_{1}}{A_{2}})\times 100\%$$

where *A*_1_ and *A*_2_ are the absorbance of the filtrated and raw solution of BSA, respectively.

#### 2.6.9. Contact Angle and Surface Energy of Membranes

Contact angle measurements of water on the wet membrane surfaces were carried out by the sessile drop method at ambient temperature using a goniometer (GBX Instruments, Berlin, Germany). Membrane samples, 3 × 3 cm^2^, were washed thoroughly with water and patted with blotting paper to remove the moisture content prior to the experiment. The sessile drop was formed on the membrane surface by depositing 5 μL water slowly and steadily with a micro syringe. The contact angle was measured at membrane-water-air interphase at room temperature within 10 s of the addition of water drop. For each sample, measurements were performed in six locations and the average was used. From the contact angle measurements, surface energy (ω_A_) was calculated as follows:

ω_A_ = γ_W_ (1 + cosθ) (4)

where γ_W_ is the surface tension (7.28 × 10^−2^ N/m).

#### 2.6.10. Molecular Weight Cut-off (MWCO)

The molecular weight cut-off (MWCO) of membranes was determined through permeation tests using 1 g/L polyethylene glycol (PEG) aqueous solution. The same concentration of PEG aqueous solution with different molecular weights of 1.2 × 10^4^, 2.0 × 10^4^, 2.6 × 10^4^, 3.0 × 10^4^, 3.6 × 10^4^, 4.0 × 10^4^ and 5.0 × 10^4^ Da were selected to perform the permeation tests. As PEG permeated solution was transparent within the scope of the ultraviolet, the concentration of PEG permeated solution was obtained by a UV–Vis spectrophotometer (UV-1801, BFRL, Beijing, China). For the points, a rejection of 90% on the rejection curves of membranes allowed the determination of the corresponding MWCO.

In order to determine membrane surface porosity, membranes were immersed in water for 4 h at 25 °C. Membrane in wet state was weighed in an electronic balance after carefully wiping the surface with a clean tissue, *W*_w_. This wet membrane was dried in an oven at 50–60 °C for 24 h. Then, membrane is weighed again in dry state, *W*_d_. The membrane surface porosity was calculated using the following equation [22]:

(5) $$Pr=\frac{W_{w}\;-\;W_{d}}{d\;\times \;A\;\times \;D}\;\times 100\%$$

where *W*_w_ is weight of wet membranes (g), *W*_d_ is weight of dry membranes (g), d is density of pure water at room temperature (1 g/cm^3^ ), D is thickness of membrane (0.02 cm) and A is area of membrane (4 cm^2^).

Mean pore radius *r* (m), on the other hand, was determined using the filtration velocity method. According to the equation, *r* could be experimentally determined by [23]:

(6) $$r={\left[\frac{8\times \left(2.9-1.75\times Pr\right)\times \eta \times D\times F}{3600\times Pr\times \Delta P}\right]}^{\frac{1}{2}}$$

where η is the water viscosity (8.9 × 10^−4^ Pa·s), D is the membrane thickness (2 × 10^-4^ m), *P*r is the membrane porosity, *F* is pure water flux (L/(m^2^·h)), and Δ*P* is the operational pressure (Pa).

## 3. Results

### 3.1. Morphology Observations of CNF, LCN and Membranes

#### 3.1.1. Transmission Electron Microscope of CNF and LCN

**Figure 3:**
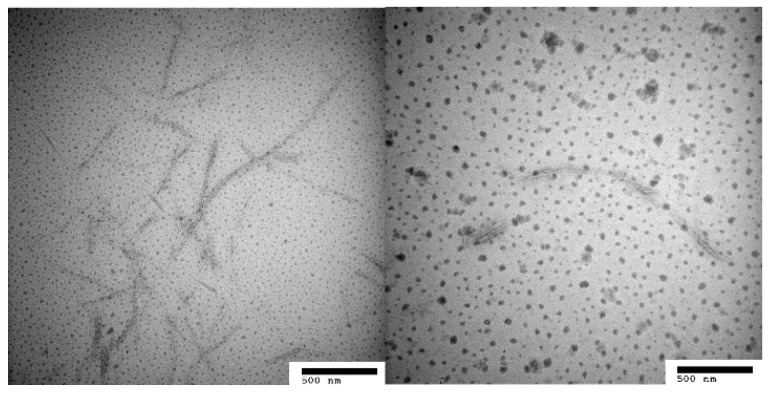
Transmission electron microscope images of CNF.

Figure 3 shows the long rod structure in the TEM image of CNF. CNF with nanosized diameters ranging from 20 to 80 nm was well-dispersed in the organic solution. Dilute sulfuric acid can break down hydrogen bonds among glucose rings (cellulose monomers) and increase the degree of crystallinity, liberating CNF into the suspension.

**Figure 4:**
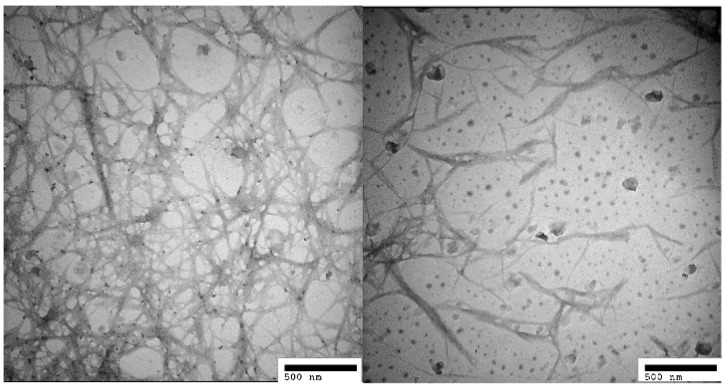
Transmission electron microscope images of LCN.

Figure 4 shows the rod-like and entanglement structure of LCN with nanosized diameters ranging from 50 to 90 nm. Dilute sulfuric acid can effectively break down the reticulated structure of lignin and the amorphous structure of cellulose, and decompose lignin–carbohydrate complexes, liberating LCN composed of lignin micromolecules and CNF into the solution. After homogenization, the LCN was distributed separately. This may be because the diluted sulfuric acid permeated into the structure of LCN and weakened the interactions among micromolecules; thus, the micro fibrils could be separated by high shear.

#### 3.1.2. Scanning Electron Microscope of Membranes

**Figure 5:**
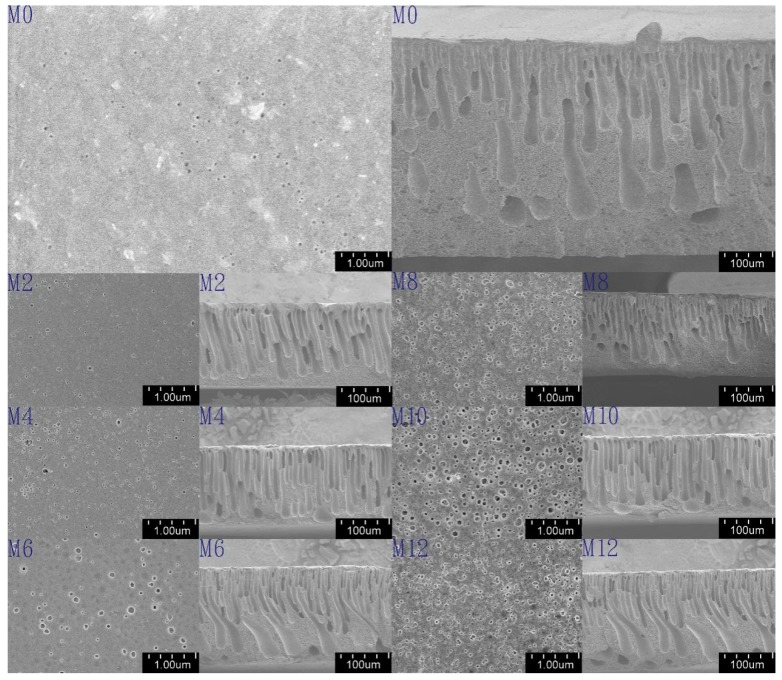
Scanning electron microscope of membranes structure.

To observe the effect of CNF and LCN on the membrane structure, SEM images of the top surface and cross-section morphologies of pure PSF and composite PSF ultrafiltration membranes are shown in Figure 5. Each cross-sectional image shows a skin layer and a support layer with tubular structures, which is expected for asymmetric ultrafiltration membranes, and thickness of membranes is about 0.2 mm. Compared with the pure PSF membrane, the tubular macrovoids were elongated across the cross-section after introducing a modification agent, CNF or LCN, and the tubular structures became wider at the bottom of the membrane. These results indicate that the mechanism of PSF membrane structure formation was not altered by the addition of CNF or LCN. The skin layer of the composites became thinner for PSF/CNF and PSF/LCN blends than that in the pure membrane, because the phase inversion process was accelerated from the large amount of hydroxyl groups exposed on the CNF and LCN surfaces [15]. Moreover, the top layer of the LCN/PSF ultrafiltration membrane was thinner and showed more connections among the top layer, sub layer and bottom layer than those in the PSF/CNF ultrafiltration membrane. Because of the absorption promoter effect of lignin on cellulose adhesion to the membrane matrix, interfacial adhesion increases the hydrogen bonds formed between PSF and cellulose [37]. The surface images of pure PSF and composite ultrafiltration membranes are shown in Figure 5. The surface pore distribution and number of surface pores on the surface of the LCN/PSF composite ultrafiltration membrane became more uniform and higher than those in the CNF/PSF composite ultrafiltration membrane. Higher hydrophilicity promotes uniform surfaces and enhances the fouling resistance of membranes. However, the pores of the blend membranes became irregular and the connections among layers became poor when excessive LCN and CNF was added because LCN and CNF easily aggregate, causing non-uniform dispersion in the casting solution [29].

### 3.2. Structure Characteristic of CNF, LCN and Membranes

#### Fourier Transform Infrared Spectroscopy

FTIR spectra of CNF, LCN, Sample M0, Sample M4 and Sample M10 were recorded to investigate the intermolecular interactions between PSF and CNF as well as that between PSF and LCN. Compared with the FTIR spectrum of the pure CNF, the spectrum of pure LCN has a broader band at 3411 cm^−1^ due to the stretching frequency of –OH group, a sharper peak at 1629 cm^−1^ ascertained to be the stretching frequency of ◎ (◎ denotes the benzene ring) and a peak at 2900 cm^−1^ that confirms the presence of C–H stretching vibrations.

The peaks corresponding to the structure of PSF polymer are as follows. Peaks at 1142, 1296 and 1321 cm^−1^ are attributed to S=O symmetric and asymmetric stretching vibrations [40,41]. The peak observed at 1100 cm^−1^ is assigned to ◎–S stretching vibration. The peaks around 700 and 1578 cm^−1^ are attributed to =C–H deformation and C=C stretching peaks, respectively [42].

**Figure 6:**
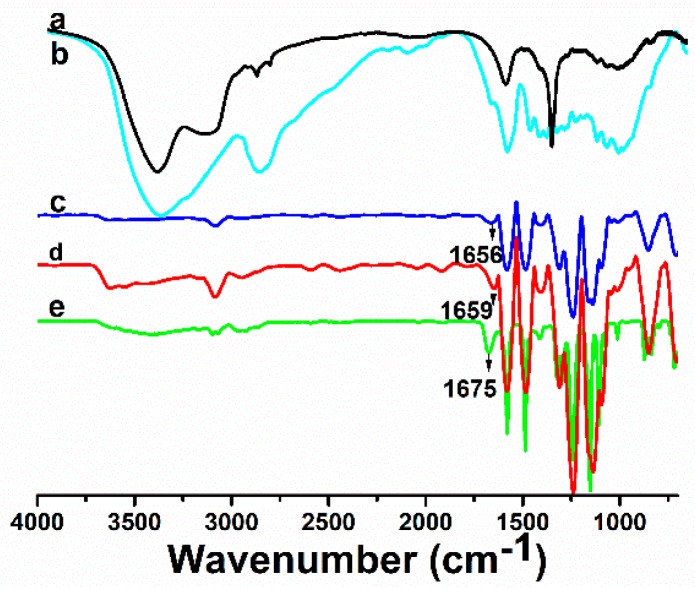
FTIR spectra of: (**a**) CNF; (**b**) LCN; (**c**) Sample M0 (PSF); (**d**) Sample M4 (CNF/PSF); and (**e**) Sample M10 (LCN/PSF).

Compared with the spectrum of the pure PSF membrane (Figure 6c), there is a new peak at 3411 cm^−1^ in Figure 6d,e, which is the characteristic peak of –OH group stretching vibrations [43]. Compared with Figure 6c, the absorption peaks of PSF/LCN ultrafiltration membrane at 1675 cm^−1^, corresponding to the stretching benzene ring stretching vibration, became broader and stronger, which indicated that hydrogen bonds were formed between PSF and CNF, and PSF and LCN.

**Figure 7:**
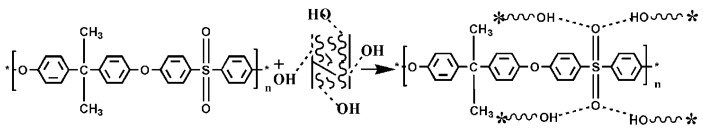
Schematic representation of the intermolecular interactions between the components in LCN/PSF ultrafiltration membranes (* denotes LCN, ~ denotes cellulose chains, and **—** denotes lignin chains).

Interactions were revealed because of the shift in the stretching frequencies of the functional groups to higher wavenumbers in the PSF/CNF and PSF/LCN ultrafiltration membranes. The spectra of Figure 6c–e indicate that the sharp band at 1675 cm^−1^, assigned to ◎ stretching vibrations, was shifted from 1656 to 1675 cm^−1^. This shift in the stretching frequencies of the PSF/LCN ultrafiltration membrane supports the establishment of hydrogen bonding between LCN and the PSF membrane matrix. The possible interaction and the established hydrogen bond between PSF and LCN as well as PSF and CNF are schematically represented in Figure 7. The schematic of such –OH···O=S=O···OH– interactions implies a good miscibility between LCN and the PSF membrane matrix. The formation of this intermolecular hydrogen bonding favors compatibility and homogeneity at the molecular scale in the PSF/LCN and PSF/CNF ultrafiltration membranes.

### 3.3. Heat Ability of CNF, LCN and Membranes

#### 3.3.1. Thermal Gravity Analysis

**Figure 8:**
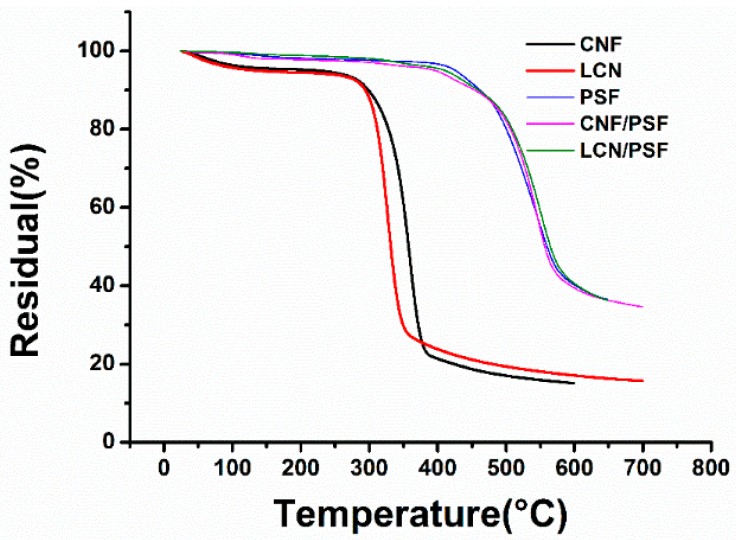
Thermal gravity analysis curves of CNF, LCN, Sample M0 (PSF), Sample M4 (CNF/PSF) and Sample M10 (LCN/PSF).

The thermal stabilities of CNF, LCN, Sample M0, Sample M4 and Sample M10 were analyzed using TGA and the results are shown in Figure 8 and Table 2. The decomposition temperature of CNF was 3.1 °C higher than that of LCN because of the higher crystallinity. In the temperature range 400 to 600 °C, the residual weight of LCN is higher than that of CNF, which can indicate the presence of hydrogen bonds between lignin and cellulose.

In the pure sample, M0, the weight loss started at 410.6 °C and continued until 600 °C because of the structure thermal stability of PSF. For Sample M4, two decomposition stages were observed at 315–424.5 °C, which was attributed to the decomposition of CNF, and 424.5–600 °C, which was attributed to the decomposition of PSF. In the temperature range from 460 to 600 °C, the residual weight of Sample M8 was more than that of Sample M3. This may be because of the hydrogen bonds formed between –OH groups of LCN and sulfone groups of PSF, indicating an absorption promoter effect of lignin on the adhesion of LCN to the PSF membrane matrix, which improves the interfacial interaction between PSF and LCN [39].

Comparing the LCN with Sample M10, the decomposition temperature of LCN in the ultrafiltration membrane was higher than that of pure LCN, which demonstrated that interactions were formed between PSF and LCN. The increase in degradation temperature indicated that the thermal stability of LCN/PSF ultrafiltration membranes improved compared with those of the pure PSF ultrafiltration membranes and CNF/PSF ultrafiltration membranes, illustrating a favorable interfacial interaction between PSF and LCN. 

#### 3.3.2. Differential Scanning Calorimetry

**Figure 9:**
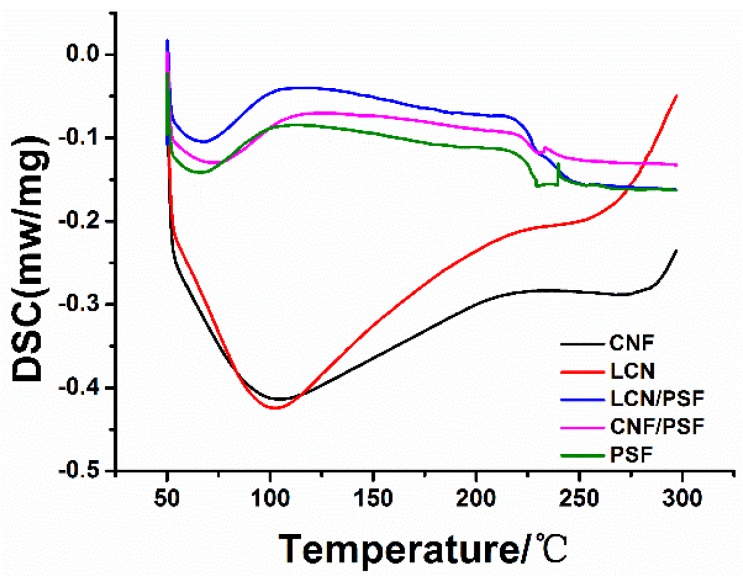
Differential scanning calorimetry curves of CNF, LCN, Sample M0 (PSF), Sample M4 (CNF/PSF) and Sample M10 (LCN/PSF).

The DSC spectra corresponding with CNF, LCN, samples of M0, M4 and M10 are shown in Figure 9. The introduction of lignin in LCN/PSF ultrafiltration membranes slightly decreased its thermal stability, probably because the addition of lignin could not enhance crystallization further, and the thermal properties of the blend membranes were slightly decreased [44]. These results suggest that miscibility between cellulose and PSF is improved because of the addition of lignin [45]. Lignin as a natural adhesion promoter improves LCN adhesion in the membrane matrix and enhances the hydrophilic and mechanical abilities of the PSF/LCN blend membranes [46].

#### 3.3.3. Rheology Analysis of Membrane Solution

Table 3 shows the shear viscosity of membrane casting solutions with different CNF and LCN concentrations. The shear viscosity of all the casting solutions was measured at 25 °C and steady-state test mode was at a shear rate of 10 s^−1^. As can be seen in Table 3, shear viscosity enhanced significantly with the increase of NCC and LCN concentration, which could be interpreted using rubberlike-liquid theory. Entanglements existed among PSF molecular chains, between cellulose molecular chains and PSF molecular chains, as well as lignin molecular chains and PSF molecular chains. As nanosized materials, CNF and LCN have large specific surface areas, which expose many hydroxyl groups on their surfaces. The hydrogen bonds between the hydroxyl groups of CNF or LCN molecules and the sulfone groups of PSF, or between the hydroxyl groups of NCC molecules themselves, create a large entanglement structure. The shear viscosity of the LCN/PSF ultrafiltration membranes was higher than that of the CNF/PSF. Interfacial adhesion form by a crosslinked network of hydrogen bonds between LCN and PSF, which enhances the entanglement density of the LCN/PSF system, causing an absorption promoter effect of lignin on LCN adhesion to the membrane matrix.

**Figure 10:**
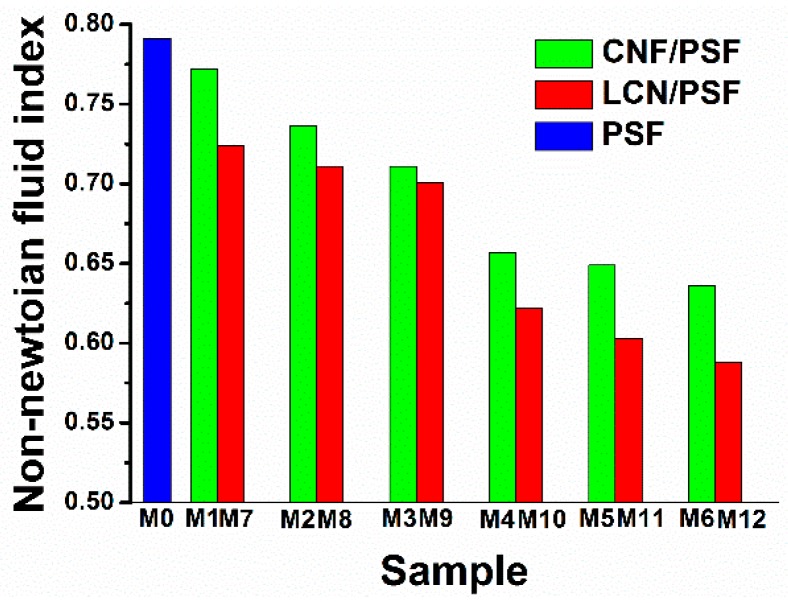
Non-Newtonian fluid index of membrane solutions: Sample M0–12.

Figure 10 shows the effects of CNF and LCN concentrations on the non-Newtonian fluid index of membrane casting solution. The non-Newtonian fluid indices decreased when the CNF or LCN concentration increased. The non-Newtonian fluid index of the LCN/PSF ultrafiltration membranes was lower than that of the CNF/PSF composites under the same conditions. Moreover, all of the non-Newtonian fluid indices were less than 1, which indicated that both the LCN/PSF and CNF/PSF were pseudoplastic fluids [47].

Following rubberlike-liquid theory, the entanglements in the membrane casting solution are connection points formed by the movements and interactions of molecular chains. When the polymer concentration increased, the average number of entanglements in every single molecule increases. Therefore, the motion of molecular chains overcomes a high resistance and energy is stored among the entanglements. The solution shows an elastic effect and deviates from Newtonian fluids [48].

#### 3.3.4. Contact Angle and Surface Energy of Membranes

**Figure 11:**
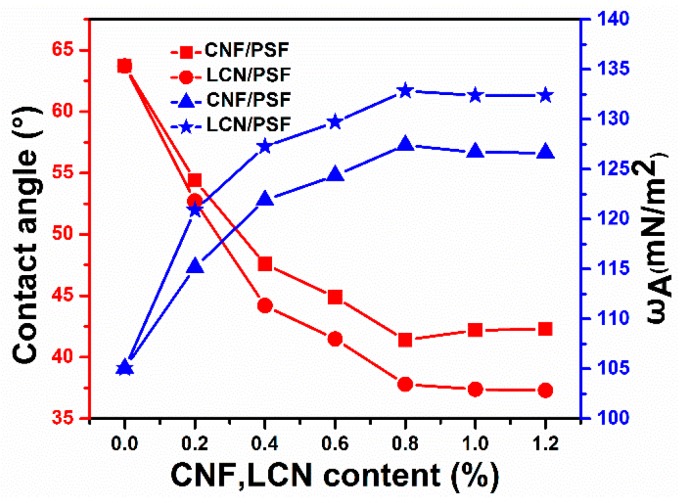
Effect of CNF and LCN contents on contact angle and surface energy of membranes.

The surface hydrophilicity has a significant effect on the water flux and membrane fouling [49]. To investigate the surface hydrophilicity and anti-fouling ability of PSF membranes, the angle between a small droplet of water and the flat horizontal surface of the membrane was measured, and the surface energy was calculated. A low contact angle represents a high tendency for water to wet the membrane, a high surface energy and high hydrophilicity [50,51]. The effect of CNF and LCN of different concentrations on the contact angle and surface energy of hybrid membranes are shown in Figure 11. The contact angle of membranes decreased and surface energy increased with increasing CNF concentrations, indicating increasing hydrophilicity of the composite membranes. The equilibrium contact angle of water decreased from 63.5° on the PSF membrane to 42.1° on the CNF/PSF membrane when the content of CNF was 0.8 wt %. CNF/PSF ultrafiltration membranes showed that the presence of polar functional groups on the surface effectively interacted with water by hydrogen bonding and recorded lower contact angles compared with that of the pure membrane. In addition to the hydroxyl functional groups, the surface porosity also showed an effect on the contact angle, because the water drop could penetrate into the pores because of the capillary action, which decreases the contact angle. The contact angle of the LCN/PSF membrane decreased and the surface energy increased more obviously than those of the CNF/PSF membranes for equivalent concentrations. The results show the effect of the lignin as a chain extender on the compatibility between cellulose and the PSF matrix. More hydrogen bonds formed on the membrane surface, improving membrane hydrophilicity [52]. After the hybrid membranes were immersed in water for 8 h, the contact angles were retained, suggesting stable hydrophilicity of the ultrafiltration membranes.

### 3.4. Mechanical Properties of Membranes

**Figure 12:**
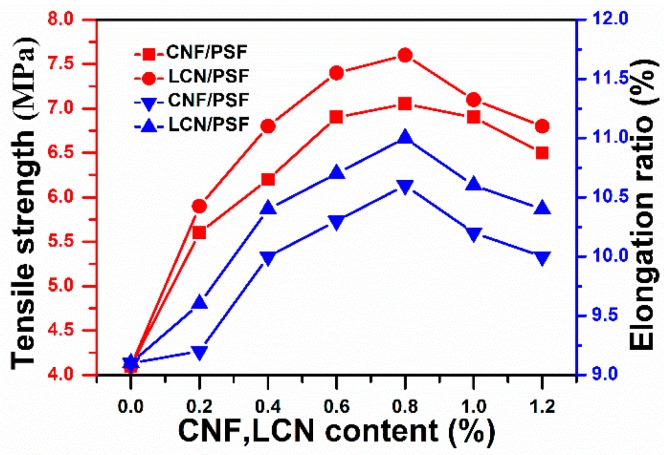
Effects of the CNF and LCN content on the tensile strength and breaking elongation of the membranes.

The tensile strength, elongation ratio and Young’s modulus are three important parameters to describe the mechanical properties of membranes. Figure 12 and Table 4 depict the tensile strengths, elongation ratios and Young’s moduli of the ultrafiltration membranes. The tensile strength, breaking elongation and Young’s modulus of CNF/PSF ultrafiltration membranes firstly increased with the addition of CNF and slightly decreased when the content was higher than 0.8 wt %, which was the weight percent with the highest mechanical properties. CNF has a high ratio of exposed hydroxyl groups relative to its dimension and a large surface area. Thus, CNF possesses a high reactive activation and surface energies. Interfacial adhesion between CNF and PSF and interfacial compatibility were enhanced because of a crosslinked network of hydrogen bonds. Therefore, CNF was distributed homogenously in PSF, and the compatibility was considered to influence the mechanical properties. However, excess CNF would aggregate and disperse non-uniformly in the PSF membrane matrix, decreasing the interfacial strength and mechanical properties of the membranes [53].

However, the ultimate tensile strength of LCN/PES ultrafiltration membranes increased by 5.6% from 7.2 to 7.6 MPa, the maximum breaking elongation enhanced by 2.8% from 10.7% to 11% and the highest Young’s moduli were also increased by 11.3% from 741 to 825 MPa compared with those of CNF/PSF ultrafiltration membranes. These observations indicate that the presence of lignin improves compatibility of the PSF matrix and cellulose, achieving a better connection between fiber and matrix than that without lignin. Lignin may be accessible for chemical reactions [52]. This finding indicates that a large proportion of the active groups at the lignin surfaces can react with the –OH groups of cellulose and the sulfone groups of PSF [54].

The tensile strength, breaking elongation and Young’s modulus of the LCN/PSF ultrafiltration membranes slightly decreased when the content was higher than 0.8 wt %, which achieved the highest mechanical properties. Cellulose nanofibrils easily aggregate and dispersed non-uniformly in the PSF membrane matrix at content levels above 0.8 wt %, decreasing interfacial strength. Lignin decreased the crosslink density of the matrix material because of a plasticized effect when LCN was added excessively, which led to a decline of mechanical properties of the membranes [53,54,55].

### 3.5. PWF, MWCO, Bovine Serum Albumin Retention Rejection and Pore Size of Membranes

**Figure 13:**
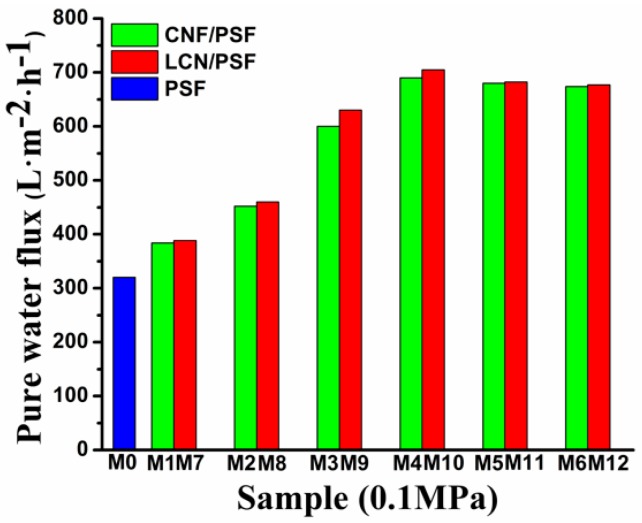
Pure water flux of membranes.

**Figure 14:**
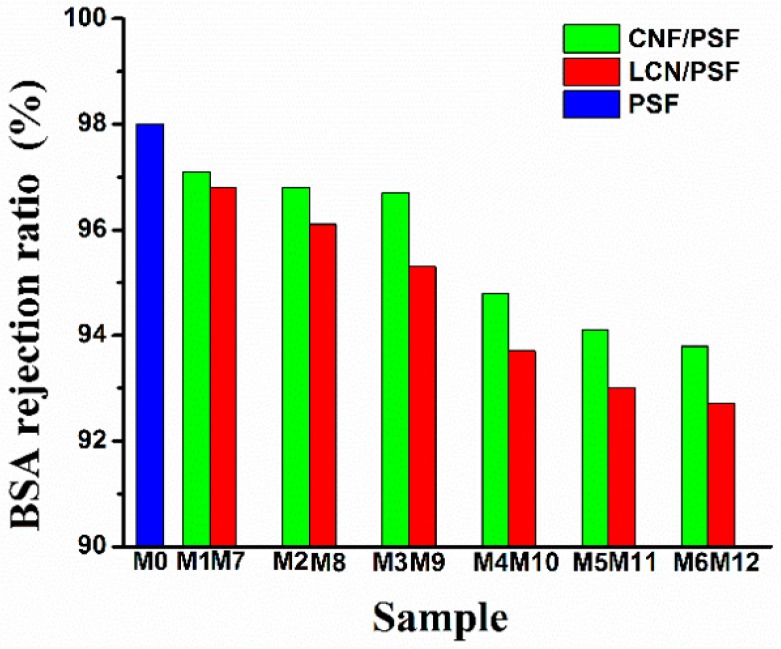
Bovine serum albumin retention rejection ratio of membranes.

PWF and BSA retention ratio are important properties for ultrafiltration membranes. The effect of CNF and LCN of different concentrations on the PWF and BSA rejection ratio are shown in Figure 13 and Figure 14, respectively. The PWF of membranes increased with the increasing dosages of CNF and LCN compared with that of the pure PSF membrane. The PWF of hybrid membranes reached 723 L/m^2^/h (with LCN), and 687 L/m^2^/h (with CNF), whereas the PWF of pure membrane was 319 L/m^2^/h. The BSA rejection ratios of the membranes slightly decreased with increasing dosages of CNF and LCN compared with that of the pure membrane, retaining a high level from 92.9% to 97.1% with or without lignin.

Generally, the water flux is influenced by the trade-off between two factors. One is the morphology, such as the pore size, distribution and thickness of skin layers and sub layers, and the other is the hydrophilicity of the membrane surface and matrix, which is related to the hydrophilic additives entrapped in the blend membranes [54].

Therefore, the flux increase in the composite membranes can be explained by the hydrophilic additives forming interconnected tubular pores and enhancing the membranes of hydrophilicity, which facilitates water molecules to pass through the membrane. The CNF contains a high ratio of exposed hydroxyl groups relative to its dimension and large surface area, showing that CNF has a high moisture absorption, which facilitates the diffusion of water into the casting solution. Therefore, trigger the instantaneous phase separation process. Furthermore, these observations indicate that the presence of lignin improves the compatibility of the PSF matrix with cellulose fibers because of its amphiphilic characteristic [56]. The absorption promoter effect of lignin on cellulose adhesion to the membrane matrix increases the number of hydrogen bonds formed between the –OH groups of cellulose and sulfone groups of PSF and enhances the PWF of the composites membrane.

The nanosized hydrophilic additives easily aggregate and disperse non-uniformly in the PSF membrane matrix when CNF and LCN are added excessively, which can cause a decrease in the interfacial strength, decreasing the PWF of the membranes [52,53,54].

**Figure 15:**
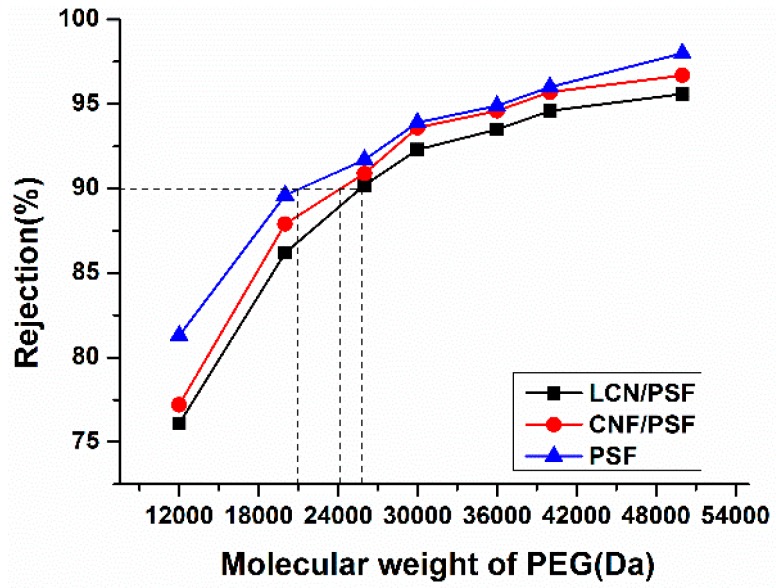
Polyethylene glycol (PEG) retention rate as a function of molecular weight for Membranes: M0, M4, and M10.

The molecular weight cut-off (MWCO) of the prepared membrane was determined through permeation tests using a group of PEG with molecular weights of 1.2 × 10^4^, 2.0 × 10^4^, 2.6 × 10^4^, 3.0 × 10^4^, 3.6 × 10^4^, 4.0 × 10^4^ and 5.0 × 10^4^ Da as model solutes. The retention rates at 0.1 MPa for the seven different PEG fractions are presented in Figure 15. From the rejection behavior, it is found that the MWCO of the hybrid membranes are about 24,169 and 25,823 Da. From the data listed in Table 5, it is shown that membranes show porosity increase by 73.7% for CNF/PSF membrane and 80.4% for LCN/PSF membrane as compared to that of pristine PSF. The pore size of membranes is about 89.1 to 150.6 nm, which indicate the characteristic of ultrafiltration membranes.

## 4. Conclusions and Discussion

In the present investigation, PSF, CNF/PSF, and LCN/PSF membranes were prepared via the immersion precipitation method by blending CNF and LCN nanoparticles in the PSF casting solution. Additionally, the compatibility, hydrophilicity and mechanical properties of LCN/PSF membranes are significantly enhanced in comparison with that of PSF membranes. The lowest equilibrium contact angle of water decreased by 8.2% from 42.1° on the CNF/PSF membrane to 33.9° on the LCN/PSF membrane. The PWF of hybrid membranes reached 723 L/m^2^/h (with LCN), and 687 L/m^2^/h (with CNF). This study could provide a new route for the fabrication of high performance ultrafiltration membrane.

Overall results suggest that membrane morphology, compatibility, mechanical property and hydrophilicity of the LCN/PSF membranes were improved significantly by the incorporation of LCN. Therefore, LCN, which eliminates the need for costly and wasteful chemical processing, should be considered as an effective modification agent for the development of ultrafiltration membranes with high hydrophilic and mechanical property.

## Figures and Tables

**Table 1:** Composition of casting solution.

Membranes	PSF (wt %)	CNF (wt %)	LCN (wt %)	PVP (wt %)	DMAC (wt %)
M0	18	-	-	1	81
M1	18	0.2	-	1	80.8
M2	18	0.4	-	1	80.6
M3	18	0.6	-	1	80.4
M4	18	0.8	-	1	80.2
M5	18	1.0	-	1	80
M6	18	1.2	-	1	79.8
M7	18	-	0.2	1	80.8
M8	18	-	0.4	1	80.6
M9	18	-	0.6	1	80.4
M10	18	-	0.8	1	80.2
M11	18	-	1.0	1	80
M12	18	-	1.2	1	79.8

**Table 2:** *T*_onset_ and *T*_max_ analysis of CNF, LCN, PSF, CNF/PSF and LCN/PSF.

Sample	CNF	LCN	PSF	CNF/PSF	LCN/PSF
***T*_onset_/°C**	274.3	271.2	410.6	424.5	431.7
***T*_max_/°C**	339.2	357.7	541.3	546.9	549.1

**Table 3:** Shear viscosity of membrane solutions.

Sample	*T* (min)									
	10	20	30	40	50	60	70	80	90	100
	Shear viscosity of the dope solutions (Pa·s)									
M0	13.61	13.59	13.58	13.59	13.58	13.58	13.57	13.55	13.54	13.53
M1	14.23	14.19	14.17	14.16	14.16	14.13	14.15	14.14	14.13	14.13
M7	15.87	15.88	15.79	15.82	15.78	15.78	15.76	15.78	15.78	15.79
M2	14.61	14.59	14.57	14.55	14.54	14.51	14.52	14.52	14.53	14.52
M8	15.41	15.37	15.36	15.38	15.39	15.47	15.43	15.39	15.39	15.39
M3	15.23	15.21	15.23	15.23	15.18	15.17	15.17	15.16	15.17	15.17
M9	17.81	17.82	17.86	17.81	17.83	17.82	17.81	17.79	17.80	17.82
M4	18.12	18.11	18.07	18.06	18.05	18.06	18.07	18.07	18.08	18.07
M10	23.52	23.50	23.51	23.52	23.51	23.50	23.51	23.52	23.53	23.51
M5	19.71	19.72	19.71	19.73	19.69	19.77	19.68	19.67	19.69	19.69
M11	25.91	25.86	25.81	25.79	25.88	25.87	25.79	25.77	25.85	25.86
M6	21.33	21.32	21.31	21.31	21.32	21.32	21.29	21.28	21.29	21.28
M12	28.79	28.84	28.59	28.80	28.67	28.86	28.74	28.51	28.75	28.76

**Table 4:** Young’s modulus of membranes (MPa).

**Sample**	**M0**	**M1**	**M2**	**M3**	**M4**	**M5**	**M6**
**Young’s modulus**	157 ± 13.16	191 ± 9.17	237 ± 17.39	310 ± 11.44	391 ± 12.17	217 ± 8.31	140 ± 12.73
**Sample**	**M7**	**M8**	**M9**	**M10**	**M11**	**M12**	
**Young’s modulus**	210 ± 11.64	271 ± 13.78	343 ± 9.45	425 ± 17.19	375 ± 8.17	291 ± 6.55	

**Table 5:** Molecular weight cut-off (MWCO), porosity and pore size of the membranes: M0, M4, and M10.

Sample	M0	M4	M10
**MWCO (Da)**	20,870	24,169	25,823
**Porosity (%)**	57.1	73.7	80.4
**Pore size (nm)**	89.1 ± 0.2	103.7 ± 0.5	150.6 ± 0.5

## References

[1] Thakur V.K., Voicu S.I. (2016). Recent advances in cellulose and chitosan based membranes forwater purification: A concise review. Carbohydr. Polym..

[2] Susanto H., Ulbrichta M. (2009). Dextran fouling of polyrsulfone ultrafiltration membranes—Causes, extent and consequences. J. Membr. Sci..

[3] Gong G., Wang J.H. (2013). Process to fabricate a new type of uniform and thin organosilica coating on polysulfone film. Mater. Lett..

[4] Shen J., Ruan H., Wu L. (2011). Preparation and characterization of PS Fulfo or gaicty using variation in CBT and addition of tetronic. J. Appl. Polym. Sci..

[5] Li J.F., Xu Z.L., Yang H., Yu L.-Y., Liu M. (2009). Effect of TiO_2_ nanoparticles on the surface morphology and performance of microporous PSF membrane. Appl. Surf. Sci..

[6] Huang J., Arthanareeswaran G., Zhang K. (2012). Effect of silver loaded sodium zirconium phosphate (nano AgZ) nanoparticles incorporation on PES membrane performance. Desalination.

[7] Chen Y., Zhang Y., Liu J. (2012). Preparation and antibacterial property of polyrsulfone ultrafiltration hybrid membrane containing halloysite nanotubes loaded with copper ions. Chem. Eng. J..

[8] Yu L., Zhang Y., Zhang B., Liu J., Zhang H., Song C. (2013). Preparation and characterization of HPEI-GO/PSF ultrafiltration membrane with antifouling and antibacterial properties. J. Membr. Sci..

[9] Miculescu M., Thakur V.K., Miculescu F., Voicu S.I. (2016). Graphene-based polymer nano composite membranes: A review. Polym. Adv. Technol..

[10] Zhang Y., Shan L., Tu Z. (2007). Preparation and characterization of novel Ce-doped nonstoichiometric nanosilica/polysulfone composite membranes. Sep. Purif. Technol..

[11] Kull R., Steen M., Fisher E. (2005). Surface modification with nitrogen-containing plasmas to produce hydrophilic, low-foulingmembranes. J. Membr. Sci..

[12] Lin J., Ye W., Zhong K. (2015). Enhancement of polyrsulfone (PSF) membrane doped by monodisperse Stöber silica for water treatment. Chem. Eng. Process..

[13] Trussell R.S., Merlo R.P., Hermanowicz S.W., Jenkins D. (2006). The effect of organic loading on process performance and membrane fouling in a submerged membrane bioreactor treating municipal wastewater. Water Res..

[14] Razi F., Sawada I., Ohmukai Y., Maruyama T., Matsuyama H. (2012). The improvement of antibiofouling efficiency of polyrsulfone membrane by functionalization with zwitterionic monomers. J. Membr. Sci..

[15] Ivnitsky H., Katz I., Minz D., Shimoni E., Chen Y., Tarchitzky J., Semiat R., Dosoretz C.G. (2005). Characterization of membrane biofouling in nanofiltration processes of wastewater treatment. Desalination.

[16] Faraji M., Yamini M., Rezaee M. (2007). Magnetic nanoparticles: Synthesis, stabilization, functionalization, characterization, and applications. Iran Chem. Soc..

[17] Zhang M., Liao B., Zhou X. (2015). Effects of hydrophilicity/hydrophobicity of membrane on membrane fouling in a submerged membrane bioreactor. Bioresour. Technol..

[18] Corobea M.C., Muhulet O., Miculescu F. (2016). Novel nanocomposite membranes from cellulose acetate and clay-silica nanowires. Polym. Adv. Technol..

[19] Dang J., Zhang Y., Du Z., Zhang H., Liu J. (2012). Antibacterial properties of PSF/CuCl_2_ three-bore hollow fiber UF membrane. Water Sci. Technol..

[20] Chen Y., Dang J., Zhang Y., Zhang H., Liu J. (2013). Preparation and antibacterial property of PSF/AgNO_3_ three-bore hollow fiber ultrafiltration membranes. Water Sci. Technol..

[21] Fan Z., Wang Z., Sun N., Wang J., Wang S. (2008). Performance improvement of polysulfone ultrafiltration membrane by blending with polyaniline nano fibers. J. Membr. Sci..

[22] Wang M., Wu L.G., Mo J.X., Gao C.-J. (2006). The preparation and characterization of novel charged polyacrylonitrile/PSF-C blend membranes used for ultrafiltration. J. Membr. Sci..

[23] Jennifer R., Sigrid P., Peter M. (2015). The preparation and characterization of novel charged poly acrylonitrile/PSF-C blend membranes used for ultrafiltration. Membr. Sci..

[24] Wang X., Cui X., Hang L. (2014). Preparation and characterization of lignin-containing nanofibrillar cellulose. Procedia Environ. Sci..

[25] Lannoy C., Soyer E., Wiesner M. (2013). Optimizing carbon nanotube-reinforced polysulfone ultrafiltration membranes through carboxylic acid functionalization. J. Membr. Sci..

[26] Li S., Gao Y., Bai H.L. (2011). Preparation and characteristics of polysulfone dialysis composite membranes modified with nanocrystalline cellulose. Bioresources.

[27] Majumdar P., Lee E., Gubbins N., Stafslien S.J., Daniels J., Thorson C.J., Chisholm B.J. (2009). Synthesis and antimicrobial activity of quaternary ammonium-functionalized POSS (Q-POSS) and polysiloxane coating containing Q-POSS. Polymer.

[28] Grainer Y., Ali A., Zaidi S. (2012). Novel sulfonated poly(ether ether ketone)/phosphonated polysulfone polymer blends for proton conducting membranes. J. Mater. Res..

[29] Beg M.D.H. (2012). Mechanical performance of Kraft fibre reinforced polypropylene composites: Influence of fibre length, fibre beating and hygrothermal ageing. J. Membr. Sci..

[30] Hancock F., Fagan S., Ziolo M. (2000). Preparation and characterization of nanoporous polysulfone membranes with high hydrophilic property using variation in CBT and addition of tetronic-1107 surfactant. Biomaterials.

[31] Gorshkova T., Kozlova L., Mikshina P. (2003). Spatial structure of plant cell wall polysaccharides and its functional significance. Biochemistry.

[32] Benavente J., Vázquez M., Hierrezuelo J. (2010). Effect of lipid nanoparticles inclusion on transport parameters through regenerated cellulose membranes. J. Membr. Sci..

[33] Vázquez M., Romero V., Hierrezuelo J. (2009). Study of ionic and diffusive transport through a regenerated cellulose nanoporous membrane. J. Membr. Sci..

[34] Jiang G., Huang G., Wang B. (2012). The changes of crystalline structure of cellulose during dissolution in 1-butyl-3-methylimidazolium chloride. Cellulose.

[35] Raghavendra G., Jayaramudu T., Varaprasad K. (2014). Microbial resistant nanocurcumin-gelatin-cellulose fibers for advanced medical applications. RSC Adv..

[36] Li X., Liu G., Liu Y. (2008). Cellulose derivatives and graft copolymers as blocks for functional materials. J. Polym. Sci..

[37] Ding Z., Zhong L., Wang X., Zhang L. (2016). Effect of lignin-cellulose nanofibrils (LCN) on the hydrophilicity and mechanical properties of polyethersulfone (PES) ultrafiltration membranes. High Perform. Polym..

[38] Zhong L., Gao Y., Li B. (2015). Preparation of hydrophilic polysulfone porous membrane by use of amphiphilic cellulose. J. Appl. Polym. Sci..

[39] Magnenet C., Jurin F.E., Lakard S., Buron C.C., Lakard B. (2013). Polyelectrolyte modification of ultrafiltration membrane for removal of copper ions. Colloids Surf. A.

[40] Rezvani-Boroujeni A., Javanbakht M., Karimi M., Shahrjerdi C., Akbariadergani B. (2015). Immobilization of thiol-functionalized nanosilica on the surface of poly(ether sulfone) membranes for the removal of heavy-metal ions from industrial wastewater samples. Ind. Eng. Chem. Res..

[41] Dai J., Yang H., Yan H., Shuangguan Y., Zheng Q., Cheng R. (2011). Phosphate adsorption from aqueous solutions by disused adsorbents: Chitosan hydrogel beads after the removal of copper(II). Chem. Eng. J..

[42] Graupner N. (2008). Application of lignin as natural adhesion promoter in cotton fibre-reinforced poly(lactic acid) (PLA) composites. J. Mater. Sci..

[43] Nathanael G., Luc V., Alice M. (2009). Pretreatments of natural fibers and their application as reinforcing material in polymer composites—A review. Compos. Sci. Technol..

[44] Li X.G., Amar M., Manju M. (2013). Multifunctionalized carbon nanotubes polymer composites: Properties and applications. Ind. Crop. Prod..

[45] Zhong R.J., Shung C.T., Li D.F. (2013). Reversible switching transitions of stimuli-responsive shape changing polymers. J. Mater. Chem. A.

[46] Ismail A.F., Lai P.Y. (2003). Effects of phase inversion and rheological factors on formation of defect-free and ultrathin-skinned asymmetric polysulfone membranes for gas separation. Sep. Purif. Technol..

[47] Lynam J.G., Chow G.I., Hyland P.L., Coronella C.J. (2016). Corn stover pretreatment by ionic liquid and glycerol mixtures with their density, viscosity, and thermo gravimetric properties. ACS Sustain. Chem. Eng..

[48] Liu Y., Yue X., Zhang S., Ren J., Yang L., Wang Q., Wang G. (2012). Synthesis of sulfonated polyphenylsulfone as candidates for antifouling ultrafiltration membrane. Sep. Purif. Technol..

[49] Xia M., Liu Q., Zhou Z., Tao Y., Li M., Liu K., Wu Z., Wang D. (2014). A novel hierarchically structured and highly hydrophilic poly(vinylalcohol-co-ethylene)/poly(ethylene terephthalate) nanoporous membrane for lithium-ion battery separator. J. Power Sources.

[50] Wang Y.M., Zhu J.Y., Dong G.Y. (2015). Sulfonated halloysite nanotubes/polyethersulfone nanocomposite membrane for efficient dye purification. Sep. Purif. Technol..

[51] Zhang C., Wu H., Michael R.K. (2015). High bio-content polyurethane composites with urethane modified lignin as filler. J. Appl. Polym. Sci..

[52] Lai S., Jing H., Li X. (2016). Phosphate adsorption by a Cu(II)-loaded polyethersulfone-type metal affinity membrane with the presence of coexistent ions. Chem. Eng. J..

[53] Qu P., Tang H., Gao Y. (2010). Polyrsulfone composite membrane blended with cellulose fibrils. Bioresources.

[54] Han M.J., Nam S.T. (2002). Thermodynamic and rheological variation in polysulfone solution by PVP and its effect in the preparation of phase inversion membrane. J. Membr. Sci..

[55] Feng Y., Wang K., Chris H.J., Wang H. (2015). Carbon nanotube/alumina/polyethersulfone hybrid hollow fiber membranes with enhanced mechanical and anti-fouling properties. Nanomaterials.

[56] Sivasankarapillai G., McDonald A.G. (2011). Synthesis and properties of lignin-highly branched poly(ester-amine) polymeric systems. Biomass Bioenergy.

